# SNPs associated with barley resistance to isolates of *Pyrenophora teres* f. *teres*

**DOI:** 10.1186/s12864-019-5623-3

**Published:** 2019-05-08

**Authors:** Irina V. Rozanova, Nina M. Lashina, Zakhar S. Mustafin, Sofia A. Gorobets, Vadim M. Efimov, Olga S. Afanasenko, Elena K. Khlestkina

**Affiliations:** 1 Institute of Cytology and Genetics, Siberian Branch of the Russian Academy of Sciences, Lavrentjeva Ave. 10, Novosibirsk, 630090 Russia; 2 N.I. Vavilov All-Russian Research Institute of Plant Genetic Resources (VIR), St. Petersburg, 190000 Russia; 3 All-Russian Research Institute for Plant Protection, St. Petersburg, 196608 Russia; 4 Novosibirsk State University, Pirogova, 1, Novosibirsk, 630090 Russia

**Keywords:** Association mapping, Barley, GWAS, *Hordeum vulgare*, Net blotch, Resistance, SNP

## Abstract

**Background:**

Net blotch caused by *Pyrenophra teres* f. *teres* is a major foliar disease of barley. Infection can result in significant yield losses of susceptible cultivars of up to 40%. Of the two forms of net blotch (*P. teres* f. *teres* and *P. teres* f. *maculata*), *P. teres* f. *teres* (net form of net blotch) is the dominant one in Russia. The goal of the current study was to identify genomic regions associated with seedling resistance to several pathotypes of the net form of net blotch in Siberian spring barley genotypes. For this, a genome-wide association study of a Siberian barley collection, genotyped with 50 K Illumina SNP-chip, was carried out.

**Results:**

Seedling resistance of 94 spring barley cultivars and lines to four *Pyrenophora teres* f. *teres* isolates (S10.2, K5.1, P3.4.0, and A2.6.0) was investigated. According to the Tekauz rating scale, 25, 21, 14, and 14% of genotypes were highly resistant, and 19, 8, 9, and 16% of genotypes were moderate-resistant to the isolates S10.2, K5.1, P3.4.0, and A2.6.0, respectively. Eleven genotypes (Alag-Erdene, Alan-Bulag, L-259/528, Kedr, Krymchak 55, Omsky golozyorny 2, Omsky 13709, Narymchanin, Pallidum 394, Severny and Viner) were resistant to all studied isolates. Nine additional cultivars (Aley, Barkhatny, Belogorsky, Bezenchuksky 2, Emelya, G-19980, Merit 57, Mestny Primorsky, Slavaynsky) were resistant to 3 of the 4 isolates. The phenotyping and genotyping data were analysed using several statistical models: GLM + Q, GLM + PCA, GLM + PCA + Q, and the MLM + kinship matrix. In total, 40 SNPs in seven genomic regions associated with net blotch resistance were revealed: the region on chromosome 1H between 57.3 and 62.8 cM associated with resistance to 2 isolates (to P3.4.0 at the significant and K5.1 at the suggestive levels), the region on chromosome 6H between 52.6 and 55.4 cM associated with resistance to 3 isolates (to P3.4.0 at the significant and K5.1 and S10.2 at the suggestive levels), three isolate-specific significant regions (P3.4.0-specific regions on chromosome 2H between 71.0 and 74.1 cM and on chromosome 3H between 12.1 and 17.4 cM, and the A2.6.0-specific region on chromosome 3H between 50.9 and 54.8 cM), as well as two additional regions on chromosomes 2H (between 23.2 and 23.8 cM, resistant to S10.2) and 3 (between 135.6 and 137.5 cM resistant to K5.1) with suggestive SNPs, coinciding, however, with known net blotch resistance quantitative trait loci (QTLs) at the same regions.

**Conclusions:**

Seven genomic regions on chromosomes 1H, 2H, 3H, and 6H associated with the resistance to four *Pyrenophora teres* f. *teres* isolates were identified in a genome-wide association study of a Siberian spring barley panel. One novel isolate-specific locus on chromosome 3 between 12.1 and 17.4 cM was revealed. Other regions identified in the current study coincided with previously known loci conferring resistance to net blotch. The significant SNPs revealed in the current study can be converted to convenient PCR markers for accelerated breeding of resistant barley cultivars.

**Electronic supplementary material:**

The online version of this article (10.1186/s12864-019-5623-3) contains supplementary material, which is available to authorized users.

## Background

Net blotch, caused by *Pyrenophora teres* (anamorph: *Drechslera teres* [Sacc.] Shoem.), is a major foliar disease of barley worldwide and in Russia. The pathogen exists in two forms based on the symptoms they cause: the net form of net blotch (*P. teres* f. *teres*) and the spot form of net blotch (*P. teres* f. *maculata*). The net form of net blotch (NFNB) is the dominant form in different regions of Russia; the spot form of net blotch (SFNB) was found only in the southern part of European Russia [1]. NFNB epidemics in Northwest Russia appear with a frequency of 5 times every 10 years [2]. Infection can result in significant yield losses of up to 40% on susceptible cultivars under favourable environmental conditions [3]; also, the disease can cause reductions in the quality of barley [4].

The most cost-effective and environmentally friendly way to control the disease is the development of resistant cultivars. The success of resistance breeding relies on the genetic diversity of resistance and the availability of resistance genes in locally adapted germplasm.

The genomic regions associated with resistance of barley to *P. teres* f. *teres* have been found on all barley chromosomes [3, 5–18] using both linkage mapping in biparental mapping populations and association mapping (AM). Some QTLs provide resistance during whole ontogenesis, such as *QRpt6* on chromosome 6H, determining both seedling and adult resistance to the net form of net blotch [5]. Other QTLs appear to be either seedling- or adult-specific [19]. Among the genomic regions associated with *P. teres* f. *teres* resistance, the region on chromosome 6H is the most well studied. It is supposed that either 3 different alleles of a single locus or three closely linked resistant genes exist in this region [9].

The goal of the current study was to identify genomic regions associated with seedling resistance to several pathotypes of the net form of net blotch. For this, a genome-wide association study of a Siberian barley collection, genotyped with 50 K Illumina SNP-chip, was carried out.

## Materials and methods

### Plant material and genotyping data

The study was based on a Siberian barley panel, consisting of 94 spring cultivars and breeding lines from the ICG GenAgro collection (Novosibirsk, Russia). Half of this panel was represented by cultivars and lines developed in breeding centres located in Siberia, whereas the other half consisted of cultivars and lines maintained in the Siberian spring barley collection, but originating from other regions and countries. Genotyping data for these 94 cultivars and lines were available from our previous study [20]. Additional information on 50 K Illumina SNP-chip loci was extracted from [21] and the BARLEYMAP resource (http://floresta.eead.csic.es/barleymap).

### Pathogen isolates and culture conditions

For phenotyping, four *Pyrenophora teres* f. *teres* single conidia isolates were used: S10.2 (Finland), K5.1 (Russia, Leningrad region), P3.4.0 (Russia, Leningrad region), and A2.6.0 (Russia, Astrakhan region). Arguments for choosing certain isolates were different origins and good sporulation ability.

Propagation of the *P. teres* isolates was conducted on Czapek’s modified medium containing 0.5 g/L KH_2_PO_4_, 0.5 g/L MgSO_4_, 0.5 g/L KCl, 1.2 g/L urea, 20 g/L lactose, and 20 g/L agar. To produce inoculum, single spore cultures were grown under near ultraviolet (UV) light with a 12 h photoperiod at 18–20 °C for 14 days. Conidia were harvested by adding distilled water to the plate and scraping the agar surface with a spatula. The suspension was filtered through two layers of cheesecloth to remove fragments of mycelia. The concentration of the inoculum was adjusted to 5000 conidia per ml. The surfactant Tween 20 was added (100 μl per litre) to facilitate dispersion of the inoculum over the leaf surfaces. Inoculation was completed by spraying at a rate of approximately 0.2 ml per plant.

### Plant growing and disease assessment

Seedling resistance was evaluated in controlled conditions in a climate chamber in the All-Russian Research Institute for Plant Protection (St. Petersburg, Russia). Three seeds of each barley cultivar were sown per pot containing nutrient-supplemented peat and cultivated for 2 weeks at 20–22 °C with a photoperiod 16 h light (exposure 5000 lx)/8 h darkness in a split-plot design with three replicates. After inoculation, plants were covered with plastic bags and placed for 48 h at 20–22 °C without light. After 2 days, inoculated plants were placed at 20–22 °C with a photoperiod 16 h light (exposure 5000 lx)/8 h darkness and air humidity of 60–70% and were grown till the disease assessment. Seedling infection responses (IRs) were assessed on the second leaf 10–12 days after inoculation. *P. teres* resistance was scored by using the 10-point scale of Tekauz [22] 1–3 = highly resistant (HR); 3.1–5.0 = moderately resistant (MR); 5.1–6.9 = moderately susceptible (MS); 7.0–10.0 = high susceptible (HS).

### Population structure

The population structure was analysed using STRUCTURE v 2.3.4 [23] based on the genotypic data of a subset of 13,659 markers. Each second marker of a set of 27,319 markers previously selected by quality control [20] was taken to reduce the computing time. The number of subpopulation (k) in the panel was inferred using an admixture model with correlated allele frequencies, a burn-in period length of 5000 and 5000 Markov chain Monte Carlo (MCMC) repetitions. Independent analyses were run for each k between 1 and 32. The estimated likelihood values [LnP(D)] were compared with k using a graph to determine the optimal k.

### Association analysis

Different statistical models were tested on disease resistance scores (separately for each of four isolates) with the help of the TASSEL 5 package [24] to detect significant marker associations: (1) generalized liner model (GLM) without correction for population structure; (2) GLM + Q: GLM + Q-matrix to account for population structure; (3) GLM + PCA: GLM with a principal component analysis (PCA) to account for population structure, (4) GLM + PCA + Q; and (5) MLM + K: MLM with kinship matrix. Genotyping data for a set of 27,319 markers previously selected by quality control [20] were used in the association analysis.

To identify significant single nucleotide polymorphisms (SNPs), two corrections were used: (i) the Bonferroni correction, where the significant threshold (0.05) is divided by the total number of tests, in this case, the total number of markers (27,319), giving the threshold 1.8302*10^− 6^, and (ii) the false discovered rate (FDR) that was calculated for each isolate in each model. The suggestive level corresponded to *p* < 10^-4th^ and was considered as suggestive evidence of an association if SNPs in the model of an isolate did not exceed the threshold value.

## Results

### Phenotyping

**Fig. 1:**
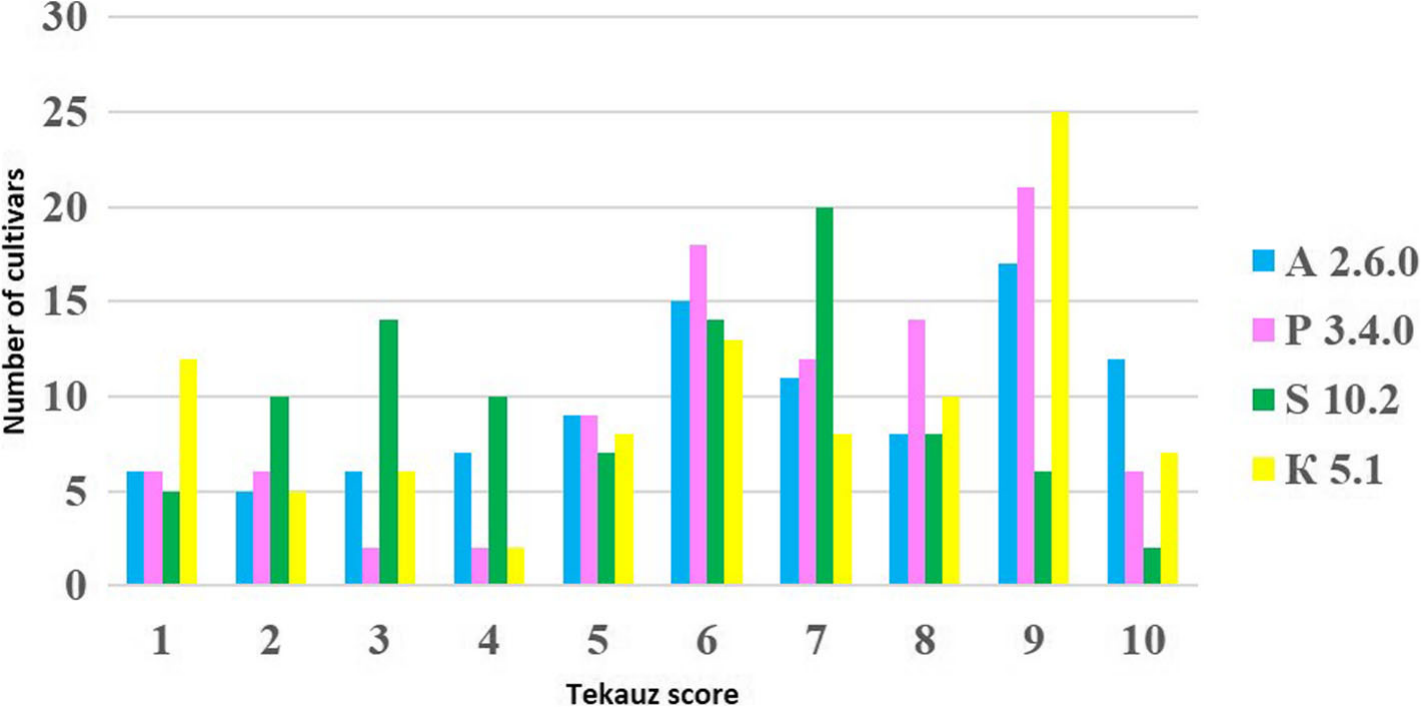
Frequency distributions for disease responses in seedling inoculations with four different *P. teres* f*.teres* isolates A 2.6.0, P 3.4.0, S10.2, K5.1

The results of the investigation of the seedling resistance to four net blotch isolates are given in Additional file 1 and summarized in Fig. 1. According to the Tekauz rating scale, 25, 21, 14, and 14% of genotypes were highly resistant and 19, 8, 9, and 16% of genotypes were moderately resistant to the isolates S10.2, K5.1, P3.4.0, and A2.6.0, respectively. Eleven genotypes (Alag-Erdene, Alan-Bulag, L-259/528, Kedr, Krymchak 55, Omsky golozyorny 2, Omsky 13709, Narymchanin, Pallidum 394, Severny and Viner) were resistant to all studied isolates. Nine additional cultivars (Aley, Barkhatny, Belogorsky, Bezenchuksky 2, Emelya, G-19980, Merit 57, Mestny Primorsky, Slavaynsky) were resistant to 3 of the 4 isolates.

### Population structure

**Fig. 2:**
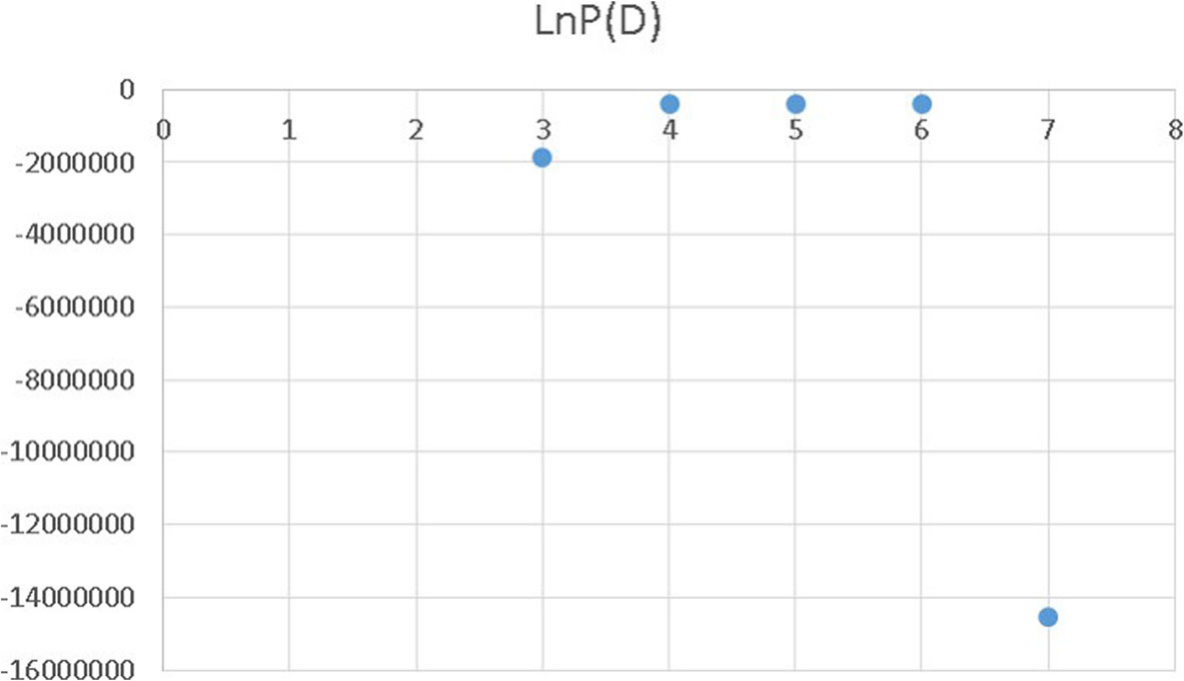
The most likely number of subpopulations was k = 4 determined by STRUCTURE v 2.3.4 The estimated likelihood values [LnP(D)] were compared with k using graph to determine the optimal k (k = 4)

**Fig. 3:**
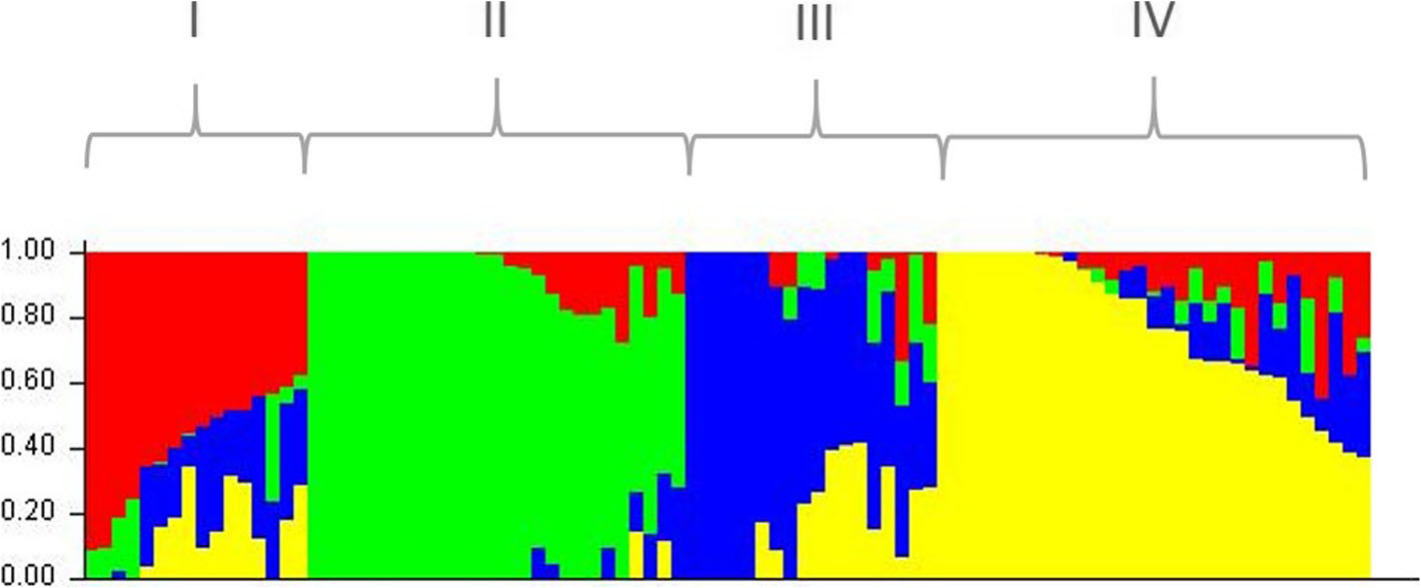
Subgrouping of barley accessions from Siberian collection based on 27,319 SNP markers. Clustering of accessions was carried out using STRUCTURE v 2.3.4 software package and Delta K value (k = 4)

The most likely number of subpopulations was k = 4 as determined by STRUCTURE v 2.3.4 (Fig. 2). The set of barley genotypes was divided into 4 groups (Fig. 3) consisting of 17, 29, 20, and 34% of genotypes. Group III contained the highest percentage of Siberian accessions (67%). Groups I, II and IV contained 31, 30 and 39% of Siberian accessions, respectively (Table 1). Percentages of highly resistant (HR), moderately resistant (MR), moderately susceptible (MS), and highly susceptible (HS) genotypes in each group is given in Table 1.

**Table 1:** Percentage content of susceptible and resistant to net blotch accessions in four clusters obtained with population structure analysis

Group	Percentage of genotypes from the total number (%)	Percentage of Siberian accessions in the group (%)	Evaluation of resistance	Isolate S10.2 (%)	Isolate K5.1 (%)	Isolate P3.4.0 (%)	Isolate A2.6.0 (%)
Group I	17	31	HR	12.5	25	19	25
			MR	31	12.5	12.5	6
			MS	31	25	31	6
			HS	25	37.5	44	32.5
Group II	29	30	HR	37	37	15	18.5
			MR	11	11	7	26
			MS	22	4	22	26
			HS	30	48	56	30
Group III	20	67	HR	11	6	6	6
			MR	22	6	11	11
			MS	17	22	22	11
			HS	50	67	67	72
Group IV	34	39	HR	29	16	16	10
			MR	19	6.5	10	16
			MS	10	26	13	26
			HS	42	52	58	48

### Genome-wide association study (GWAS) analysis

The results of all statistical models were first compared in quantile-quantile (QQ) plot to find proper models for each dataset. QQ-plots for the models used are presented for each *P. teres* f. *teres* isolate in Additional file 2. The GLM analysis without correction for population structure showed a great number of false positive SNPs in a QQ-plot (Additional file 2). A QQ-plot using the GLM model accounting for population structure (GLM + Q) appeared to be more proper; in the case of the P.3.4.0 isolate, a very good match with expected values was observed (Additional file 2). Similarly, the GLM + PCA and GLM + PCA + Q models appeared to be more proper than GLM. Association mapping results using different models are presented in Additional file 3. With the help of the GLM + Q model, two significant SNPs on chromosome 6H were revealed for the isolate P3.4.0, one significant SNP on chromosome 3H for the isolate A2.6.0, one suggestive SNP on chromosome 1H for the isolate K5.1 and two suggestive SNPs (1 SNP on chromosome 2H and 1 SNP on chromosome 5H) for isolate S10.2 (Additional file 3; Table 2).

**Table 2:** SNPs associated with resistance to the isolates A2.6.0, P3.4.0, K5.1 and S10.2, revealed by GLM analysis with accounting of population structure (GLM + Q model)

Isolate	*p*-value	Marker	Chr	Position	cM	Allele	Minor Allele	SNP associated with resistance
A2.6.0	1.01E-06	JHI-Hv50k-2016-183207^a^	3H	490244247	52.6-54.8	A/T	T(0.41)	T(0.75)
P3.4.0	8.11E-08	SCRI_RS_239642^a^	6H	357492232	55.4	A/G	A(0.22)	A(0.83)
P3.4.0	2.09E-07	SCRI_RS_224389^a^	6H	360336381	55.4	C/T	C(0.22)	C(0.83)
K5.1	5.89E-6	JHI-Hv50k-2016-33160^b^	1H	441912080	57.3-58.2	G/A	A(0.21)	G(0.95)
S10.2	1.82E-05	JHI-Hv50k-2016-74407^b^	2H	31977763	23.2-23.8	C/T	C(0.46)	T(0.68)
S10.2	2.89E-05	BOPA1_5206–787^b^	5H	1154245	NA	G/C	G(0.14)	C(1)
S10.2	4.95E-05	JHI-Hv50k-2016-391875^b^	6H	142513704	52.6–53.8	A/C	A(0.30)	C(1)
S10.2	4.95E-05	BOPA2_12_30021^b^	6H	156957594		A/G	A(0.30)	G(1)

^a^Significant SNPs according the Bonferroni correction

^b^Suggestive SNPs

The GLM analysis with PCA accounting for the population structure (GLM + PCA) revealed 2 significant SNPs on chromosome 6H and 2 significant SNPs on chromosome 2H associated with resistance to the isolate P.3.4.0. Additionally, 2 suggestive SNPs (1 SNP on chromosome 3H and 1 SNP on chromosome 6H close to the region revealed for P3.4.0 isolate) were associated with resistance to the K5.1 isolate, and 3 suggestive SNPs on chromosome 6H were associated with resistance to S10.2 (Additional file 3; Table 3).

**Table 3:** Single nucleotide polymorphism (SNP) markers associated with resistance P3.4.0, A2.6.0, S10.2 and K5.1 isolates, revealed by GLM + PCA analysis according *p*-value

Isolate	p-value	Marker	chr	Position	cM	Allele	Minor Allele	SNP associated with resistance
P3.4.0	3.24e-8	SCRI_RS_239642^a^	6H	357492232	55.4	A/G	A(0.22)	A(0.83)
P3.4.0	4.84e-8	SCRI_RS_224389^a^	6H	360336381	55.4	C/T	C(0.22)	C(0.83)
P3.4.0	7.42E-06	JHI-Hv50k-2016-104859^b^	2H	639342580	71-74.1	A/G	G(0.18)	A(0.50)
P3.4.0	1.44E-05	BOPA2_12_31445^b^	2H	639288665	71-74.1	A/G	G(0.20)	A(0.50)
A2.6.0	2.08E-06	JHI-Hv50k-2016-169338^c^	3H	213632124	50.9-52.6	A/G	G(0.40)	G(0.92)
A2.6.0	5.96E-06	SCRI_RS_186341^c^	3H	NA	51.2	G/A	A(0.40)	A(0.92)
S10.2	2.20E-06	JHI-Hv50k-2016-392656^c^	6H	153234073	52.6–53.8	C/T	C(0.48)	T(0.86)
S10.2	2.38E-06	JHI-Hv50k-2016-391875^c^	6H	142513704		A/C	A(0.30)	C(1)
S10.2	2.38E-06	BOPA2_12_30021^c^	6H	156957594		A/G	A(0.30)	G(1)
K5.1	2.84E-05	JHI-Hv50k-2016-215624^c^	3H	668951958	135.6-137-5	A/C	C(0.11)	A(1)
K5.1	3.14E-05	SCRI_RS_239642^c^	6H	357492232	55.4	A/G	A(0.22)	A(0.55)

^a^Significant SNPs according the Bonferroni correction

^b^Significant SNPs according the FDR

^c^Suggestive SNPs

The GLM analysis with a combination of two corrections GLM + PCA + Q revealed 7 significant SNPs on chromosome 6H, 7 significant SNPs on chromosome 2H, 8 SNP on chromosome 1H and 6 significant SNPs on chromosome 3H (Additional file 3; Table 4).

**Table 4:** Single nucleotide polymorphism (SNP) markers associated with resistance P3.4.0, S10.2 and K5.1 isolates, revealed by GLM + PCA + Q analysis according p-value

Isolate	p-value	Marker	chr	Position	cM	Allele	Minor Allele	SNP associated with resistance
P3.4.0	7.64E-07	SCRI_RS_239642^a^	6H	357492232	55.4	A/G	A(0.22)	A(0.83)
P3.4.0	6.72E-07	SCRI_RS_224389^a^	6H	360336381	55.4	C/T	C(0.22)	C(0.83)
P3.4.0	2.30E-07	JHI-Hv50k-2016-104859^a^	2H	639342580	71-74.1	A/G	G(0.18)	A(0.50)
P3.4.0	1.58E-06	BOPA2_12_31445^a^	2H	639288665	71-74.1	A/G	G(0.20)	A(0.50)
P3.4.0	9.13E-06	JHI-Hv50k-2016-35839^b^	1H	463250609	62.3-62.8	T/G	T(0.38)	G(0.75)
P3.4.0	1.60E-05	JHI-Hv50k-2016-398663^b^	6H	359349968	55.4	T/C	C(0.25)	C(0.83)
P3.4.0	1.60E-05	SCRI_RS_138529^b^	6H	86866524	55.4	T/C	C(0.25)	C(0.83)
P3.4.0	2.59E-05	JHI-Hv50k-2016-104508^b^	2H	638564394	71–74.1	C/T	T(0.24)	T(0.50)
P3.4.0	2.59E-05	JHI-Hv50k-2016-104565^b^	2H	638636071		C/T	T(0.24)	T(0.50)
P3.4.0	2.59E-05	JHI-Hv50k-2016-104567^b^	2H	638636277		C/T	T(0.24)	T(0.50)
P3.4.0	2.59E-05	JHI-Hv50k-2016-104667^b^	2H	638690222		G/T	T(0.24)	T(0.50)
P3.4.0	2.82E-05	JHI-Hv50k-2016-104669^b^	2H	638690416		A/C	C(0.24)	A(0.50)
P3.4.0	4.35E-05	JHI-Hv50k-2016-33086^b^	1H	438893078	57.3-58.2	C/A	A(0.26)	C(0.75)
P3.4.0	4.64E-05	JHI-Hv50k-2016-156591^b^	3H	17271039	12.1–17.4	T/C	C(0.23)	T(0.75)
P3.4.0	4.77E-05	JHI-Hv50k-2016-156539^b^	3H	16631590		G/T	T(0.14)	G(0.75)
P3.4.0	4.77E-05	JHI-Hv50k-2016-156576^b^	3H	17177586		T/C	C(0.14)	T(0.75)
P3.4.0	4.77E-05	JHI-Hv50k-2016-156584^b^	3H	17269072		C/G	C(0.14)	G(0.75)
P3.4.0	5.54E-05	JHI-Hv50k-2016-33097^b^	1H	439174674	57.3–58.2	G/A	A(0.27)	G(0.75)
P3.4.0	5.54E-05	JHI-Hv50k-2016-33109^b^	1H	439297538		G/A	A(0.27)	C(0.75)
P3.4.0	6.58E-05	JHI-Hv50k-2016-156594^b^	3H	17292680	12.1–17.4	A/T	T(0.22)	A(0.75)
P3.4.0	6.66E-05	JHI-Hv50k-2016-156586^b^	3H	17270441		T/A	T(0.23)	A(0.75)
P3.4.0	7.32E-05	JHI-Hv50k-2016-33099^b^	1H	439176975	57.3–58.2	C/T	T(0.26)	C(0.75)
P3.4.0	7.32E-05	JHI-Hv50k-2016-33104^b^	1H	439177729		T/C	C(0.26)	T(0.75)
P3.4.0	7.59E-05	JHI-Hv50k-2016-398720^b^	6H	359701468	55.4	C/T	T(0.10)	C(0.58)
P3.4.0	7.59E-05	JHI-Hv50k-2016-398886^b^	6H	361528770		C/T	C(0.10)	T(0.58)
P3.4.0	7.59E-05	JHI-Hv50k-2016-398894^b^	6H	361531190		C/T	C(0.10)	T(0.58)
P3.4.0	9.74E-05	JHI-Hv50k-2016-33120^b^	1H	439541073	57.3-58.2	C/G	G(0.27)	C(0.75)
P3.4.0	1.01E-04	JHI-Hv50k-2016-36398^b^	1H	467931082	62.3-62.8	T/G	G(0.27)	T(0.83)
S10.2	1.10E-05	JHI-Hv50k-2016-392723^c^	6H	155949390	52.6–53.8	A/G	G(0.48)	A(0.86)
S10.2	1.10E-05	JHI-Hv50k-2016-393052^c^	6H	163763506		T/A	T(0.48)	A(0.86)
S10.2	2.98E-05	JHI-Hv50k-2016-392656^c^	6H	153234073		A/G	A(0.47)	T(0.86)
S10.2	7.02E-05	JHI-Hv50k-2016-391875^c^	6H	142513704		A/C	A(0.30)	C(1)
S10.2	7.02E-05	BOPA2_12_30021^c^	6H	156957594		A/G	A(0.30)	G(1)
K5.1	1.41E-04	JHI-Hv50k-2016-215624^c^	3H	668951958	135.6-137.5	A/C	C(0.11)	A(1)
K5.1	1.45E-04	JHI-Hv50k-2016-33160^c^	1H	441912080	57.3-58.2	G/A	A(0.21)	A(0.55)

^a^Significant SNPs according the Bonferroni correction

^b^Significant SNPs according the FDR

^c^Suggestive SNPs

No significant SNP was revealed using the MLM analysis with the kinship matrix (MLM + K model).

## Discussion

The analysis of the population structure of the Siberian barley panel revealed 4 clusters. We noticed that among 14 accessions susceptible to all four net blotch isolates, 8 originated from Siberia (57.1%), and among 23 accessions susceptible to three isolates, 5 (22%) originated in Siberia.

The GWAS performed using five statistical models revealed seven genomic loci associated with resistance to one to three net blotch isolates. The comparison of these regions with locations of previously known *P. teres* resistance QTLs is presented in Table 5.

**Table 5:** Comparison of the regions associated with resistance to *P. teres* f. *teres*, identified in the current study and previous studies

chr	Isolate	p-value	Marker	cM	Previuosly identified QTLs (cM)
1H	P3.4.0	4.35E-05	JHI-Hv50k-2016-33086^b^	**57.3-62.8**	**(52.4-56.8 cM) Grewal et al., 2012** [10]**;** **(50–86 cM) Afanasenko et al., 2014** [2]**;** (92.2 cM) Amezrou et al., 2018 [8]; (95.9 cM) Vatter et al. 2017 [18]
	P3.4.0	5.54E-05	JHI-Hv50k-2016-33097^b^		
	P3.4.0	7.32E-05	JHI-Hv50k-2016-33099^b^		
	P3.4.0	7.32E-05	JHI-Hv50k-2016-33104^b^		
	P3.4.0	5.54E-05	JHI-Hv50k-2016-33109^b^		
	P3.4.0	9.74E-05	JHI-Hv50k-2016-33120^b^		
	K5.1	1.45E-04	JHI-Hv50k-2016-33160^c^		
	P3.4.0	9.13E-06	JHI-Hv50k-2016-35839^b^		
	P3.4.0	1.01E-04	JHI-Hv50k-2016-36398^b^		
2H	S10.2	1.82E-05	JHI-Hv50k-2016-74407^c^	**23.2-23.8**	**(10-28.7 cM) Wonneberger et al., 2017** [7]**;** **(8 cM, 23 cM) Vatter et al. 2017** [18]
	P3.4.0	2.59E-05	JHI-Hv50k-2016-104508^b^	**71.0-74.1**	(48 cM) Arru et al., 2003 [11]; (50–51 cM) Grewal et al., 2008 [5]; (54.2–55.4 cM) Steffenson et al., 1996 [3]; (55.5 cM) Vatter et al. 2017 [18]; (62.7 cM) Cakir et al., 2011 [26]; **(51–75 cM) Afanasenko et al., 2014** [2]; (75–80 cM) König et al., 2014 [25]; (57.15, 59.35 cM and 92.22 cM) Amezrou et al., 2018 [8]; (120.04–125.35 сМ) Richards et al., 2016 [17]
	P3.4.0	2.59E-05	JHI-Hv50k-2016-104565^b^		
	P3.4.0	2.59E-05	JHI-Hv50k-2016-104567^b^		
	P3.4.0	2.59E-05	JHI-Hv50k-2016-104667^b^		
	P3.4.0	2.82E-05	JHI-Hv50k-2016-104669^b^		
	P3.4.0	1.58E-06	BOPA2_12_31445^a^		
	P3.4.0	2.30E-07	JHI-Hv50k-2016-104859^a^		
3H	P3.4.0	4.77E-05	JHI-Hv50k-2016-156539^b^	12.1-17.4	(8.5 cM) Vatter et al. 2017 [18];
	P3.4.0	4.77E-05	JHI-Hv50k-2016-156576^b^		
	P3.4.0	4.77E-05	JHI-Hv50k-2016-156584^b^		
	P3.4.0	6.66E-05	JHI-Hv50k-2016-156586^b^		
	P3.4.0	4.64E-05	JHI-Hv50k-2016-156591^b^		
	P3.4.0	6.58E-05	JHI-Hv50k-2016-156594^b^		
	A2.6.0	2.08E-06	JHI-Hv50k-2016-169338^c^	**50.9-54.8**	**(52.6-54.8 cM) Koladia et al.,2017** [9]**;** **(51.6 cM) Vatter et al. 2017** [18]**;** **(46.2–54.5 cM) Wonneberger et al., 2017** [7]
	A2.6.0	5.96E-06	SCRI_RS_186341^c^		
	A2.6.0	1.01E-06	JHI-Hv50k-2016-183207^a^		
	K5.1	1.41E-04	JHI-Hv50k-2016-215624^c^	**135.6-137.5**	(115–119 cM) Grewal et al., 2008 [5]; **(112–150 cM) Afanasenko et al., 2014** [2]**;** **(137,6 cM) Amezrou et al., 2018** [8]
6H	P3.4.0	1.60E-05	SCRI_RS_138529^b^	**52.6-55.4**	**(50.8–66.4) Wonneberger et al., 2017** [7]**;** **(50.2–58.4 cM) Koladia et al., 2017** [9]**;** (58 cM) Afanasenko et al., 2014 [2]; (60–65 cM) König et al., 2014 [25]; (75–78 cM) Grewal et al., 2008 [5]
	S10.2	2.38E-06	JHI-Hv50k-2016-391875^c^		
	S10.2	2.20E-06	JHI-Hv50k-2016-392656^c^		
	S10.2	1.10E-05	JHI-Hv50k-2016-392723^c^		
	S10.2	2.38E-06	BOPA2_12_30021^c^		
	S10.2	1.10E-05	JHI-Hv50k-2016-393052^c^		
	P3.4.0	3.24E-08	SCRI_RS_239642^a^		
	K5.1	3.14E-05	SCRI_RS_239642^c^		
	P3.4.0	1.60E-05	JHI-Hv50k-2016-398663^b^		
	P3.4.0	7.59E-05	JHI-Hv50k-2016-398720^b^		
	P3.4.0	4.84E-08	SCRI_RS_224389^a^		
	P3.4.0	7.59E-05	JHI-Hv50k-2016-398886^b^		
	P3.4.0	7.59E-05	JHI-Hv50k-2016-398894^b^		

^a^Significant SNPs according the Bonferroni correction

^b^Significant SNPs according the FDR

^c^Suggestive SNPs

Our loci that were confirmed by literature data are in bold

### Chromosome 1 H

Nine SNPs that were detected on chromosome 1H in the current study are associated with resistance to the P3.4.0 and K5.1 isolates. They are located in the interval 57.3–62.8 cM and expanded between markers JHI-Hv50k-2016-33086 and JHI-Hv50k-2016-36398. Earlier, Grewal et al. (2012) [10] revealed locus between 52.4–56.8 cM, while Afanasenko et al. (2014) [2] reported QTLs between 50 and 86 cM on this chromosome. We suggest that seedling resistance to the newly studied isolates P3.4.0 and K5.1 is conferred in Siberian barley germplasm by the previously known locus found on chromosome 1H by Grewal et al. (2012) [10] and Afanasenko et al. (2014) [2]. In addition, chromosome 1H is known to carry another locus at approximately 40 cM from this one, described by Amezrou et al. (2018) [8] and Vatter et al. [18] (Table 5).

### Chromosome 2H

The resistance to different isolates P3.4.0 and S10.2 was associated with two different loci. The locus associated with resistance to the S10.2 isolate included one suggestive SNP (JHI-Hv50k-2016-74407) mapped in the interval 23.3–23.8 cM. We suggest that seedling resistance to the S10.2 isolate is conferred by a previously known locus mapped earlier by Wonneberger et al. (2017) [7] and Vatter et al. (2017) [18] (Table 5). The locus on chromosome 2H revealed for the P3.4.0 isolate included 7 SNPs and was expanded between JHI-Hv50k-2016-104508 and JHI-Hv50k-2016-104859 markers located in the interval 71.0–74.1 cM. This region coincides with location of the previously described QTL mapped between 51 and 75 cM by Afanasenko et al. (2014) [2]. The locus detected by König et al. (2014) [25] between 75 and 80 cM can be the same. A further known region on chromosome 2H is located between the 2 regions detected in the current study. It includes loci in the positions 48 cM [11], 50–51 cM [5]; 54.2–55.4 cM [3], 55.5 cM [18], 57.15 [8], 59.35 [8], and 62.7 cM [26]. In addition, more distal loci were found on chromosome 2H by Amezrou et al. (2018) [8] (92.22 cM) and Richards et al. (2016) [17] (120.04–125.35 сМ).

### Chromosome 3H

Three regions associated with different isolates were revealed on chromosome 3H. The locus in the interval 12.1–17.4 cM between markers JHI-Hv50k-2016-156539 and JHI-Hv50k-2016-156594 was significantly associated with resistance to the P3.4.0 isolate. The closest locus among the QTLs mapped earlier is the locus at 8.5 cM reported by Vatter et al. (2017) [18]. It is suggested that the region revealed in the current study between 12.1 and 17.4 cM may carry a novel locus not described earlier. A further region associated with markers JHI-Hv50k-2016-183207, JHI-Hv50k-2016-169338, and SCRI_RS_186341 in the interval 50.9–54.8 cM was revealed for the A2.6.0 isolate. This region was earlier reported to be associated with net blotch resistance by Koladia et al. (2017) [9] (52.6–54.8 cM), Vatter et al. (2017) [18] (51.6 cM), and Wonneberger et al. (2017) [7] (46.2–54.5 cM). JHI-Hv50k-2016-183207 is located less than 500 kb from the marker SCRI_RS_221644 reported by Koladia et al. (2017) [9]. The locus associated with resistance to the K5.1 isolate included one suggestive SNP (JHI-Hv50k-2016-215624) mapped in the interval 135.6–137.5 cM. We suggest that seedling resistance to the K5.1 isolate is conferred by a previously known locus mapped earlier by Afanasenko et al. (2014) [2] and Amezrou et al. (2018) [8] (Table 5).

### Chromosome 6H

The region on chromosome 6H between 52.6 and 55.4 cM was associated with resistance to three isolates (to P3.4.0 at the significant and K5.1 and S10.2 at the suggestive levels). Among the 12 SNPs revealed in the current study is SCRI_RS_239642 and SCRI_RS_224389, reported earlier to be associated with NFNB resistance [17, 18]. The presence of QTL for NFNB resistance in barley chromosome 6H was reported in several studies. First, this locus was found by Steffenson et al. (1996) [3], then confirmed by Manninen et al. (2006) [27] and named *RPt5*. Wonneberger et al. (2017) [7] and Afanasenko et al. (2014) [2] revealed the genomic region associated with NFNB resistance in the same interval. Further loci were revealed close to this region [5, 16] (Table 5).

## Conclusions

Seven genomic regions on chromosomes 1H, 2H, 3H, and 6H associated with the resistance to four *Pyrenophora teres* f. *teres* isolates were identified in a genome-wide association study of Siberian spring barley panel. One novel isolate-specific locus on chromosome 3 between 12.1 and 17.4 cM was revealed. Other regions identified in the current study coincided with previously known loci conferring resistance to net blotch. The significant SNPs revealed in the current study can be converted to convenient PCR-markers for accelerated breeding of resistant barley cultivars.

## Additional files

Additional file 1: Table with results of net blotch resistance assessment within the Siberian spring barley collection. HR – highly resistant (1.0–3.0); MR – moderately resistant (3.1–5.0); MS – moderately susceptible (5.1–6.9); S – susceptible (7.0–10.0); “-” – failed. (DOCX 43 kb)

Additional file 2: QQ-plots (quantile-quantile plots) for the models: (1) GLM without correction for population structure; (2) GLM + Q: GLM + Q-matrix to account for population structure; (3) GLM + PCA, (4) GLM + PCA + Q (5) MLM + K: MLM with kinship matrix. (DOCX 255 kb)

Additional file 3: Association mapping results using different models: GLM + Q (GLM + Q-matrix to account for population structure), GLM + PCA, GLM + PCA + Q, MLM + K (MLM with kinship matrix). Dash line named “Bonferroni” corresponds the Bonferroni threshold. Dash line named “FDR” corresponds the FDR (false discovered rate) threshold. (DOCX 5895 kb)

## Abbreviation

AM: Association mapping
FDR: False discovery rate
GLM: General linear model
GWAS: Genome-wide association study
HR: High resistant
HS: High susceptible
IRs: Infection responses
MLM: Mixed linear model
MR: Moderate resistant
MS: Moderate susceptible
NFNB: Net form net blotch
PCA: Principal component analysis
Q: Population structure
QQ plot: Quantile-quantile plot
QTL: Quantitative trait locus
SFNB: Spot form net blotch
SNP: Single nucleotide polymorphism
