# Supplementary material for: SNPs associated with barley resistance to isolates of Pyrenophora teres f. teres
Source: BMC Genomics. 2019 May 8;20(Suppl 3):292. doi: 10.1186/s12864-019-5623-3 (PMC7227216; doi:10.1186/s12864-019-5623-3)
Supplement: Supplementary file 1 — Table with results of net blotch resistance assessment within the Siberian spring barley collection. HR – highly resistant (1.0–3.0); MR – moderately resistant (3.1–5.0); MS – moderately susceptible (5.1–6.9); S – susceptible (7.0–10.0); “-” – failed. (DOCX 43 kb) [file 12864_2019_5623_MOESM1_ESM.docx]

**Additional file 1:** Table with results of net blotch resistance assessment within the Siberian spring barley collection. HR – highly resistant (1.0 – 3.0); MR – moderately resistant (3.1-5.0); MS – moderately susceptible (5.1-6.9); S – susceptible (7.0-10.0); “-” – failed.

| **Cultivar/line name** | **Reaction to S10.2** | | **Reaction to K5.1** | | **Reaction to P3.4.0** | | **Reaction to A2.6.0** | |
| --- | --- | --- | --- | --- | --- | --- | --- | --- |
|  | **Value** | **Rating scale** | **Value** | **Rating scale** | **Value** | **Rating scale** | **Value** | **Rating scale** |
| Abyssinia | **4.5** | **MR** | **6** | **MS** | **8** | **S** | **9** | **S** |
| AC 0760258 | **8.5** | **S** | **9.7** | **S** | **9.3** | **S** | **6.7** | **MS** |
| Acha | **7.5** | **S** | **10** | **S** | **6** | **MS** | **8.7** | **S** |
| Agul | **7.5** | **S** | **1.8** | **R** | **8.7** | **S** | **5** | **MR** |
| Alag-Erdene | **1.3** | **HR** | **1.3** | **HR** | **1.3** | **HR** | **1.3** | **HR** |
| Alan-Bulag | **3** | **HR** | **1.7** | **HR** | **1** | **HR** | **2.3** | **HR** |
| Aley | **3** | **HR** | **7** | **S** | **1.7** | **HR** | **1.3** | **HR** |
| Alyn-Buya | **2.5** | **HR** | **9** | **S** | **9.7** | **S** | **9.7** | **S** |
| Anna | **7** | **MS** | **8** | **S** | **7.7** | **S** | **6** | **MS** |
| Archekas | **8.3** | **S** | **10** | **S** | **9** | **S** | **9.7** | **S** |
| Arna | **3** | **HR** | **6.5** | **MS** | **7.3** | **S** | **6.7** | **MS** |
| Avalon | **7.8** | **S** | **8.3** | **S** | **9.3** | **S** | **9** | **S** |
| B-1 | **1.3** | **HR** | **9** | **S** | **5.7** | **MS** | **4.3** | **MR** |
| Bagan | **6** | **MS** | **5.3** | **MS** | **9** | **S** | **5** | **MR** |
| Barkhatny | **5** | **MR** | **1** | **HR** | **6** | **MS** | **3.7** | **MR** |
| Belogorsky | **6.7** | **MS** | **1.5** | **HR** | **1.3** | **HR** | **3.7** | **MR** |
| Bezenchuksky 2 | **5** | **MR** | **1.5** | **HR** | **5** | **MR** | **8** | **S** |
| Biom | **4.8** | **MR** | **7.3** | **S** | **9.3** | **S** | **9.3** | **S** |
| Brachny | **7** | **S** | **7** | **S** | **6** | **MS** | **7.7** | **S** |
| Chelyabinsky 70 | **7** | **S** | **2.5** | **HR** | **10** | **S** | **10** | **S** |
| Dobry | **7** | **S** | **10** | **S** | **8.7** | **S** | **9.7** | **S** |
| Donetsky 8 | **1.3** | **S** | **1.3** | **HR** | **8.3** | **S** | **6** | **MS** |
| Emelya | **1.3** | **HR** | **5** | **MR** | **7.7** | **S** | **5** | **MR** |
| G-19951 | **8** | **S** | **6** | **MS** | **7.7** | **S** | **9** | **S** |
| G-19980 | **3** | **HR** | **6** | **MS** | **4** | **MR** | **5** | **MR** |
| G-21038 | **6.3** | **MS** | **8.3** | **S** | **10** | **S** | **9.3** | **S** |
| G-21219 | **6.8** | **MS** | **9.7** | **S** | **6,3** | **MS** | **9** | **S** |
| G-21671 | **8.8** | **S** | **5** | **MR** | **6** | **MS** | **7.7** | **S** |
| G-21672 | **7** | **S** | **9** | **S** | **8.7** | **S** | **10** | **S** |
| Golozyorny 1 | **3.3** | **MR** | **10** | **S** | **7** | **S** | **8** | **S** |
| Granal | **6.5** | **MS** | **2.3** | **HR** | **5.3** | **MS** | **4.3** | **MR** |
| Ilmen | **2.3** | **HR** | **9** | **S** | **7** | **S** | **4.3** | **MR** |
| Impuls | **9.8** | **S** | **7** | **MS** | **6** | **MS** | **9.7** | **S** |
| Jngve | **10** | **S** | **10** | **S** | **4.7** | **MR** | **6.7** | **MS** |
| Kedr | **2.5** | **HR** | **2** | **HR** | **2.7** | **HR** | **4** | **MR** |
| Kolchan | **1.5** | **HR** | **5.5** | **MS** | **8** | **S** | **6.7** | **MS** |
| Krasnoyarsky 1 | **7** | **S** | **10** | **S** | **9.7** | **S** | **9.5** | **S** |
| Krasnoyarsky 91 | **7.3** | **S** | **6.8** | **MS** | **7.5** | **S** | **5.3** | **MS** |
| Krymchak 55 | **4** | **MR** | **4.5** | **MR** | **5** | **MR** | **4** | **MR** |
| Kuryer | **9.8** | **S** | **9.5** | **S** | **7** | **S** | **8.7** | **S** |
| L-1 | **6.5** | **MS** | **5.7** | **MS** | **5.5** | **MS** | **7.7** | **S** |
| L-1285 | **6.5** | **MS** | **1.7** | **HR** | **9** | **S** | **8.3** | **S** |
| L-259/528 | **2** | **HR** | **2.8** | **HR** | **2.5** | **HR** | **1** | **HR** |
| L-421 | **9** | **S** | **9** | **S** | **6** | **MS** | **1.7** | **HR** |
| Manych 459 | **6.8** | **MS** | **10** | **S** | **8.7** | **S** | **9.7** | **S** |
| Mayak | **7.3** | **S** | **9.5** | **S** | **8.7** | **S** | **8.7** | **S** |
| Medikum | **7** | **S** | **4.3** | **MR** | **2** | **HR** | **7.5** | **S** |
| Melius | **7.3** | **S** | **9.3** | **S** | **9.3** | **S** | **10** | **S** |
| Merit 57 | **3.5** | **MR** | **5.5** | **MS** | **2.3** | **HR** | **3.3** | **MR** |
| Mestny Dagestanian | **5.7** | **MS** | **6** | **MS** | **2** | **HR** | **2** | **HR** |
| Mestny Ethiopian | **4** | **MR** | **9** | **S** | **5** | **MR** | **8** | **S** |
| Mestny Primorsky | **6** | **MS** | **2** | **HR** | **1.3** | **HR** | **1** | **HR** |
| Mestny Yakutian | **2** | **HR** | **8.5** | **S** | **8,5** | **S** | **7** | **S** |
| Moskovsky 121 | **5.3** | **MS** | **7** | **S** | **9.7** | **S** | **9.7** | **S** |
| Mutant 68 | **3** | **HR** | **6.3** | **MS** | **6.7** | **MS** | **7.3** | **S** |
| Narymchanin | **4.5** | **MR** | **1.5** | **HR** | **3.7** | **MR** | **2.5** | **HR** |
| NGB 112412 | **5** | **MR** | **8.5** | **S** | **6.5** | **MS** | **10** | **S** |
| Nikita | **6** | **MS** | **9.8** | **S** | **7.3** | **S** | **7.7** | **S** |
| Nosovsky 11 | **2.5** | **HR** | **9.5** | **S** | **9.7** | **S** | **10** | **S** |
| Novosibirsky 80 | **3** | **HR** | **9.5** | **S** | **10** | **S** | **6.5** | **MS** |
| Nutans 274 | **7** | **S** | **9** | **S** | **9.7** | **S** | **9.3** | **S** |
| Nutans 970 | **8.7** | **S** | **8.7** | **S** | **10** | **S** | **6.3** | **MS** |
| Obskoy | **9.3** | **S** | **6** | **MS** | **6,7** | **MS** | **5.3** | **MS** |
| Omsky 13709 | **1.7** | **HR** | **1.3** | **HR** | **1.3** | **HR** | **1.3** | **HR** |
| Omsky 85 | **6** | **MS** | **3** | **HR** | **6.3** | **MS** | **2.5** | **HR** |
| Omsky golozyorny 1 | **7.3** | **S** | **6.5** | **MS** | **9.7** | **S** | **10** | **S** |
| Omsky golozyorny 2 | **3** | **HR** | **3** | **HR** | **2** | **HR** | **3** | **HR** |
| Orenburgsky kormovoy | **7** | **S** | **9.3** | **S** | **9.5** | **S** | **6.3** | **MS** |
| Oskar | **9.5** | **S** | **9.7** | **S** | **9** | **S** | **10** | **S** |
| Pallidum 394 | **3.8** | **MR** | **3.8** | **MR** | **2.7** | **HR** | **2.3** | **HR** |
| Priekulsky 14 | **2.3** | **HR** | **9** | **S** | **8** | **S** | **3** | **HR** |
| Reyd | **3.5** | **MR** | **6** | **MS** | **6.3** | **MS** | **6** | **MS** |
| Sasha | **5.3** | **MS** | **9.8** | **S** | **8** | **S** | **6.7** | **MS** |
| Selection from Tyal | **6.5** | **MS** | **9.3** | **S** | **9.7** | **S** | **5.5** | **MS** |
| Severny | **2.3** | **HR** | **1** | **HR** | **5** | **MR** | **4.7** | **MR** |
| Signal | **7.8** | **S** | **6.7** | **MS** | **7** | **S** | **9.7** | **S** |
| Simvol | **10** | **S** | **3** | **HR** | **10** | **S** | **10** | **S** |
| Slavyansky | **3.3** | **MR** | **9** | **S** | **5** | **MR** | **4.7** | **MR** |
| Sobolyok | **7.3** | **S** | **8** | **S** | **9.3** | **S** | **7** | **S** |
| Svetik | **2** | **HR** | **5** | **MR** | **7** | **S** | **7.5** | **S** |
| Symbat | **4** | **MR** | **6.7** | **MS** | **6.5** | **MS** | **6.7** | **MS** |
| Taganay | **8.3** | **S** | **9** | **S** | **8.7** | **S** | **8.3** | **S** |
| Talan | **8** | **S** | **10** | **S** | **9.7** | **S** | **10** | **S** |
| Tanay | **9.5** | **S** | **7.3** | **MS** | **8.3** | **S** | **6.3** | **MS** |
| Tarsky 1 | **8.8** | **S** | **3.5** | **MR** | **6.3** | **MS** | **7** | **S** |
| Tatum | **6.3** | **MS** | **9.5** | **S** | **10** | **S** | **10** | **S** |
| Temp | **7** | **MS** | **5.3** | **MS** | **8.7** | **S** | **10** | **S** |
| Vikont | **4.5** | **MR** | **10** | **S** | **9.7** | **S** | **9.3** | **S** |
| Viner | **3** | **HR** | **1** | **HR** | **3.2** | **MR** | **3.5** | **MR** |
| Viner mutant | **6.5** | **MS** | **3.3** | **MR** | **7** | **S** | **6** | **MS** |
| Vorsinsky 2 | **4** | **MR** | **8** | **S** | **9.3** | **S** | **10** | **S** |
| Vybor | **4.5** | **MR** | **9.5** | **S** | **5** | **MR** | **9.3** | **S** |
| Wial | **3** | **HR** | **8.5** | **S** | **9.3** | **S** | **7** | **S** |
| Zalarinets | **2.8** | **HR** | **7.5** | **S** | **6.3** | **MS** | **5.3** | **MS** |
| Zernogradsky 86 | **9** | **S** | **7** | **S** | **7** | **S** | **6** | **MS** |
| Zolotnik | **4.7** | **MR** | **6** | **MS** | **6** | **MS** | **5** | **MR** |
| **Harrington** | **9.8** | **S** | **9.8** | **S** | **10** | **S** | **10** | **S** |
| **(control)** |  |  |  |  |  |  |  |  |
