# Supplementary material for: SNPs associated with barley resistance to isolates of Pyrenophora teres f. teres
Source: BMC Genomics. 2019 May 8;20(Suppl 3):292. doi: 10.1186/s12864-019-5623-3 (PMC7227216; doi:10.1186/s12864-019-5623-3)

**Additional file 2:** QQ-plots (quantile-quantile plots) for the models: (1) GLM without correction for population structure; (2) GLM + Q: GLM + Q-matrix to account for population structure; (3) GLM + PCA, (4) GLM + PCA + Q (5) MLM + K: MLM with kinship matrix.

**QQ-plot for GLM model without population structure:**


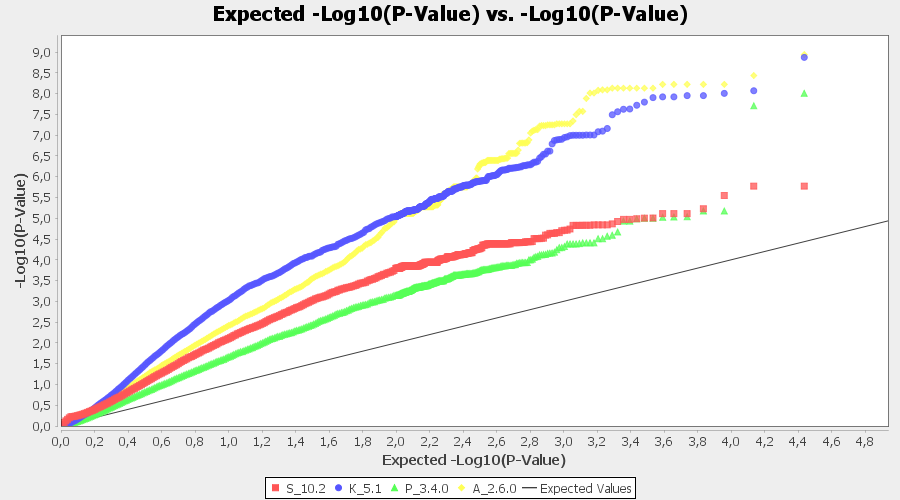


**QQ-plot for GLM+Q model (GLM plot with accounting of population structure):**


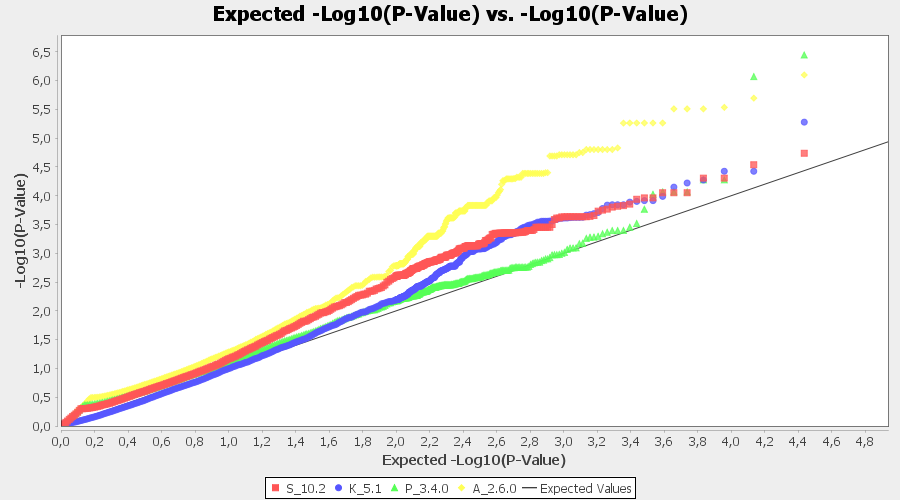


**QQ-plot for GLM+PCA model:**


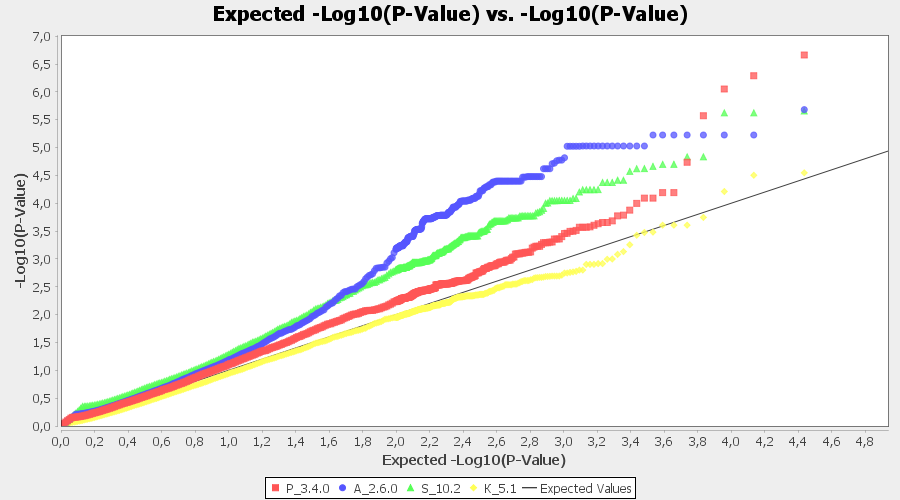


**QQ-plot for GLM+PCA+Q model:**


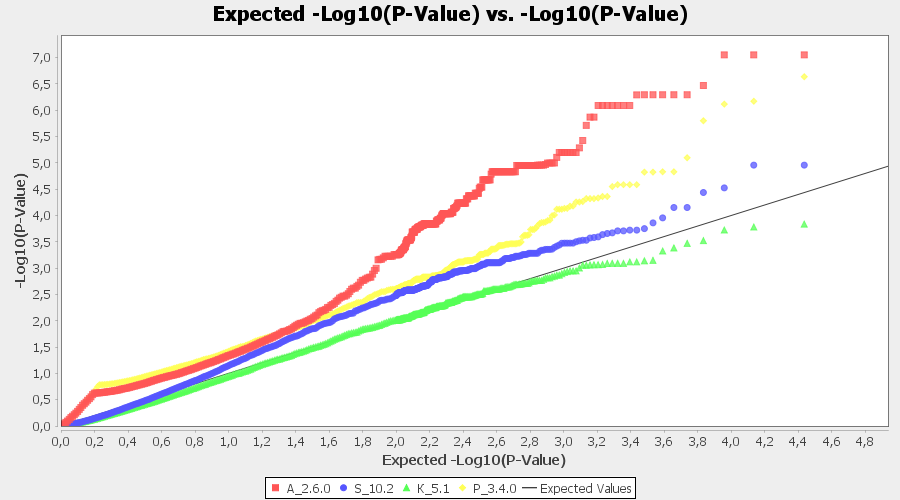


**QQ-plot for MLM + K model:**


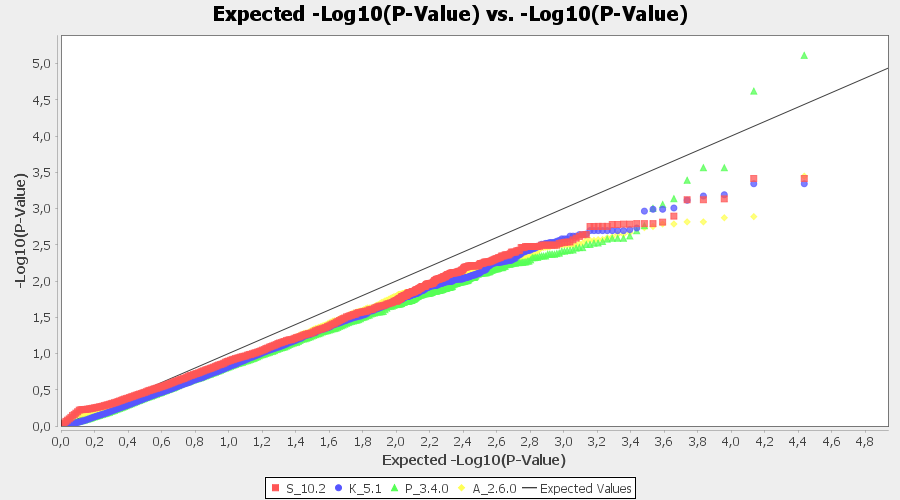

Supplement: Supplementary file 2 — QQ-plots (quantile-quantile plots) for the models: (1) GLM without correction for population structure; (2) GLM + Q: GLM + Q-matrix to account for population structure; (3) GLM + PCA, (4) GLM + PCA + Q (5) MLM + K: MLM with kinship matrix. (DOCX 255 kb) [file 12864_2019_5623_MOESM2_ESM.docx]
