# Supplementary material for: SNPs associated with barley resistance to isolates of Pyrenophora teres f. teres
Source: BMC Genomics. 2019 May 8;20(Suppl 3):292. doi: 10.1186/s12864-019-5623-3 (PMC7227216; doi:10.1186/s12864-019-5623-3)

**Additional file 3:** Association mapping results using different models: GLM + Q (GLM + Q-matrix to account for population structure), GLM + PCA, GLM + PCA + Q, MLM + K (MLM with kinship matrix). Dash line named “Bonferroni” corresponds the Bonferroni threshold. Dash line named “FDR” corresponds the FDR (false discovered rate) threshold.

**GLM+Q model for the isolate A2.6.0:**


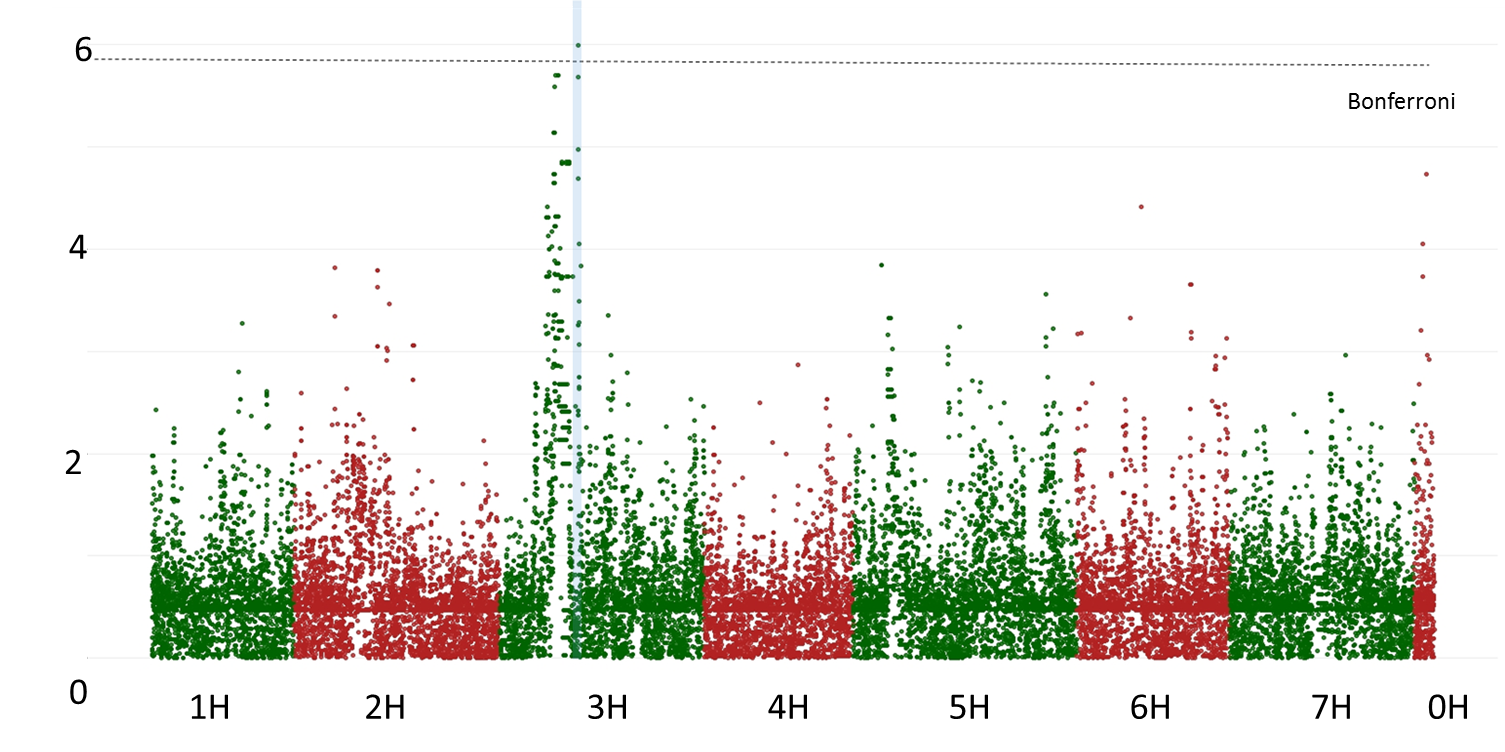


**GLM+Q model for the isolate K5.1:**


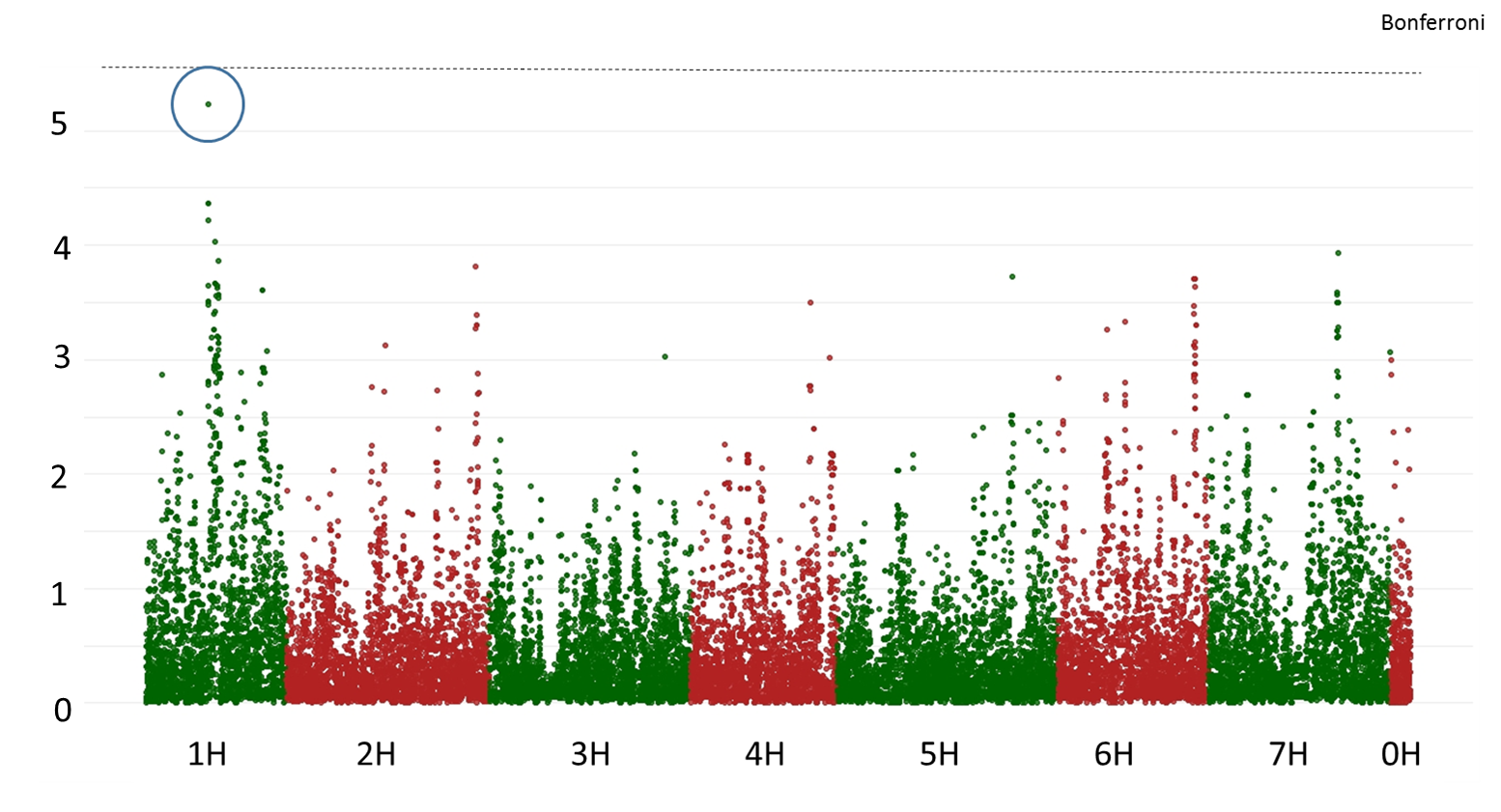


**GLM+Q model for the isolate P3.4.0:**

**
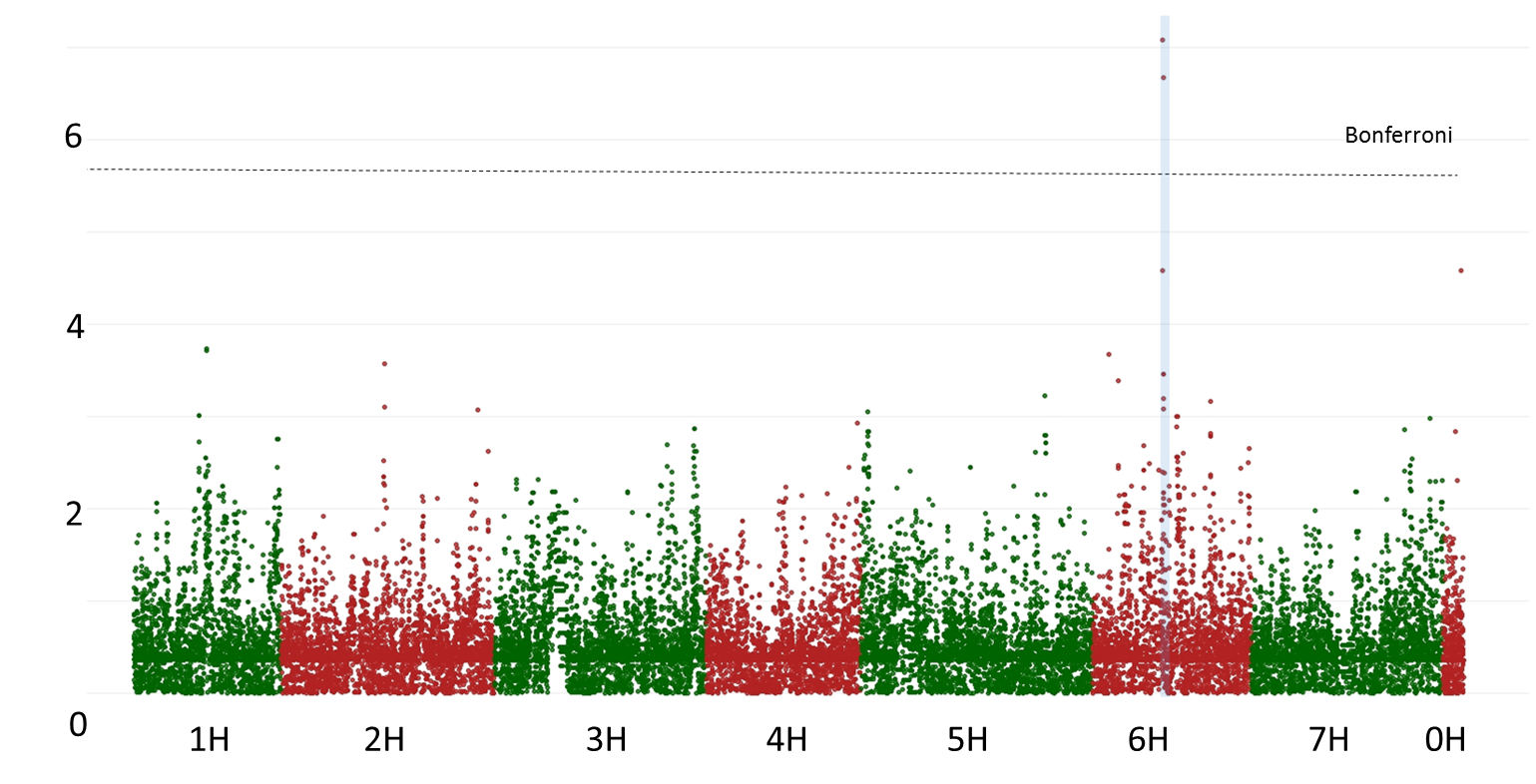
**

**GLM+Q model for the isolate S10.2:**

**
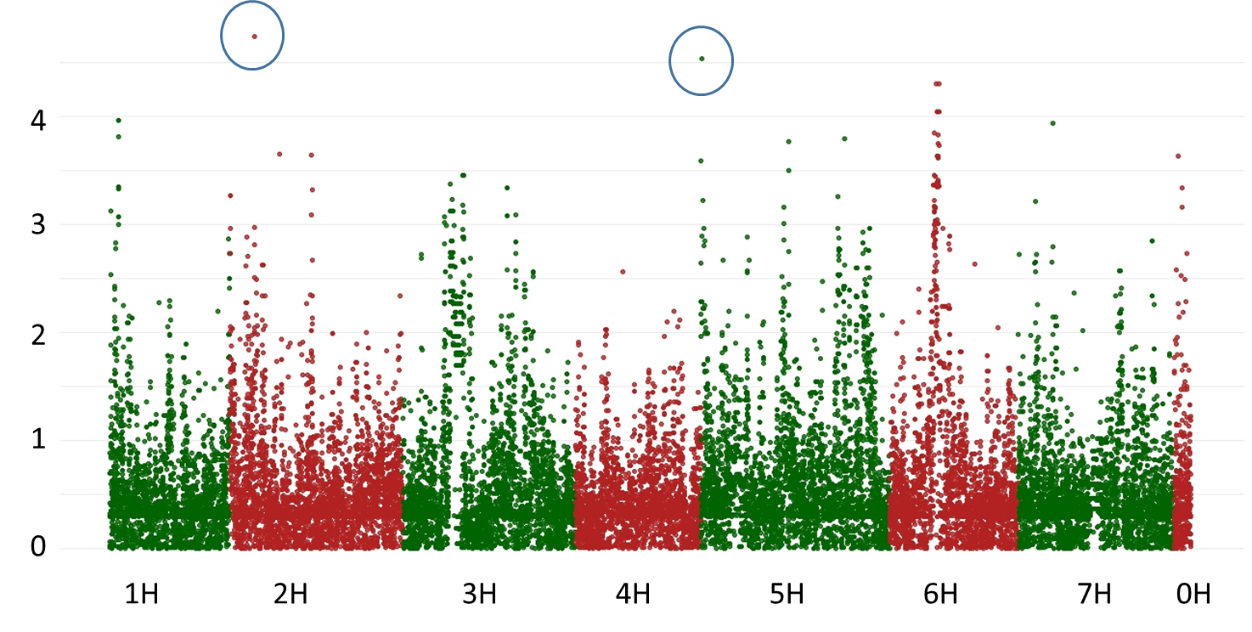
**

**GLM+PCA model for the isolate P3.4.0:**


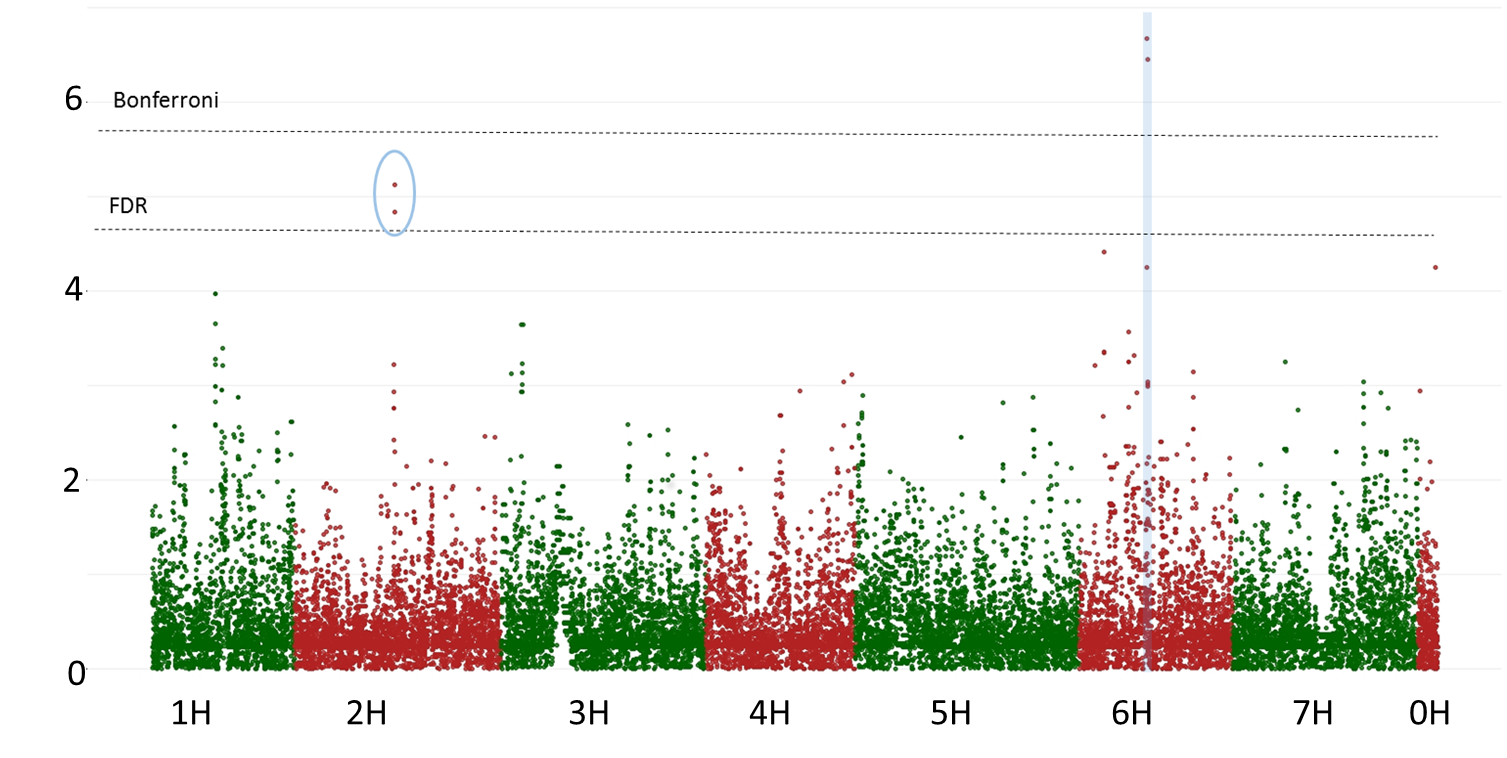


**GLM+PCA model for the isolate K5.1:**


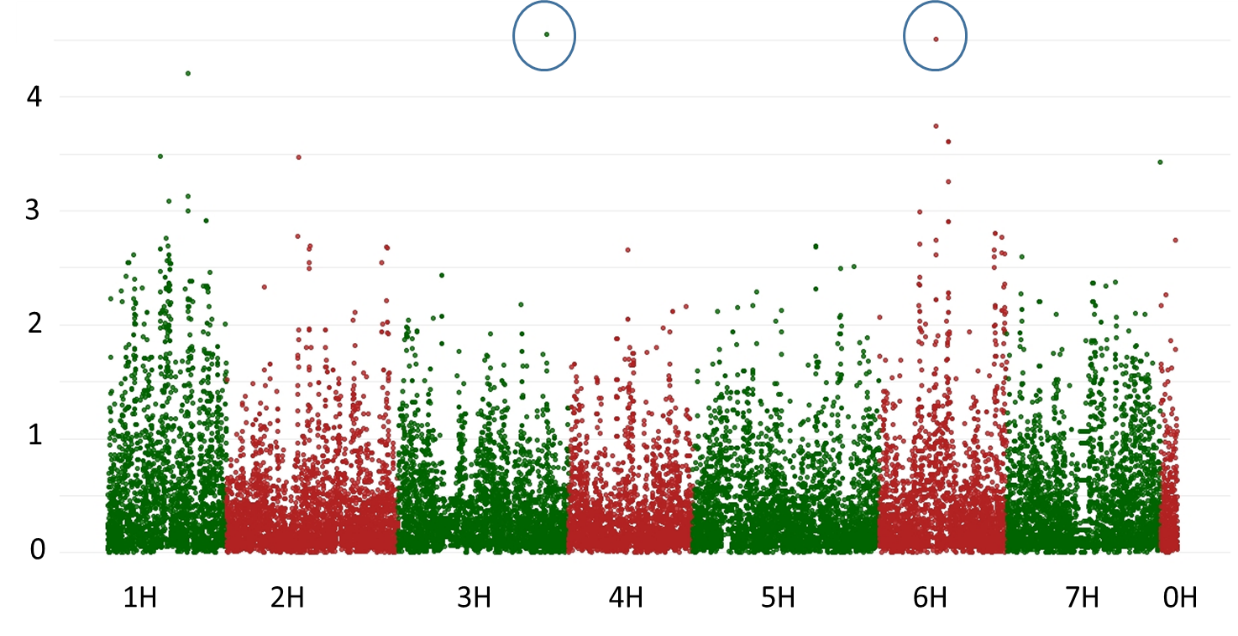


**GLM+PCA model for the isolate S10.2:**


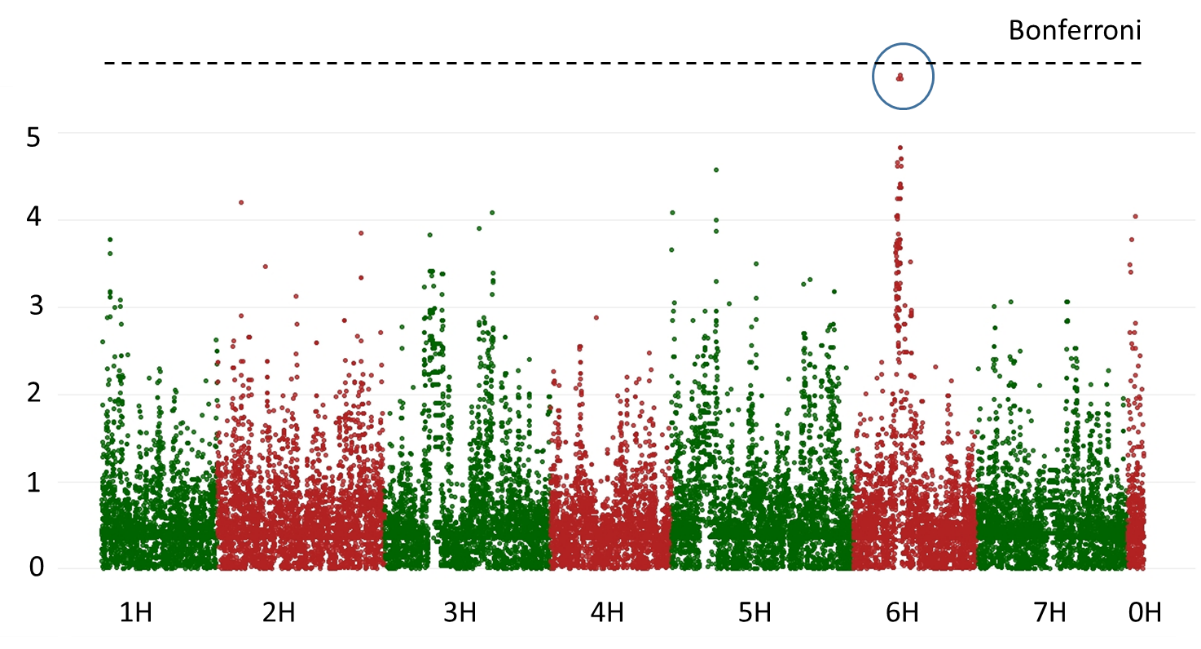


**GLM+PCA+Q model for the isolate P3.4.0:**


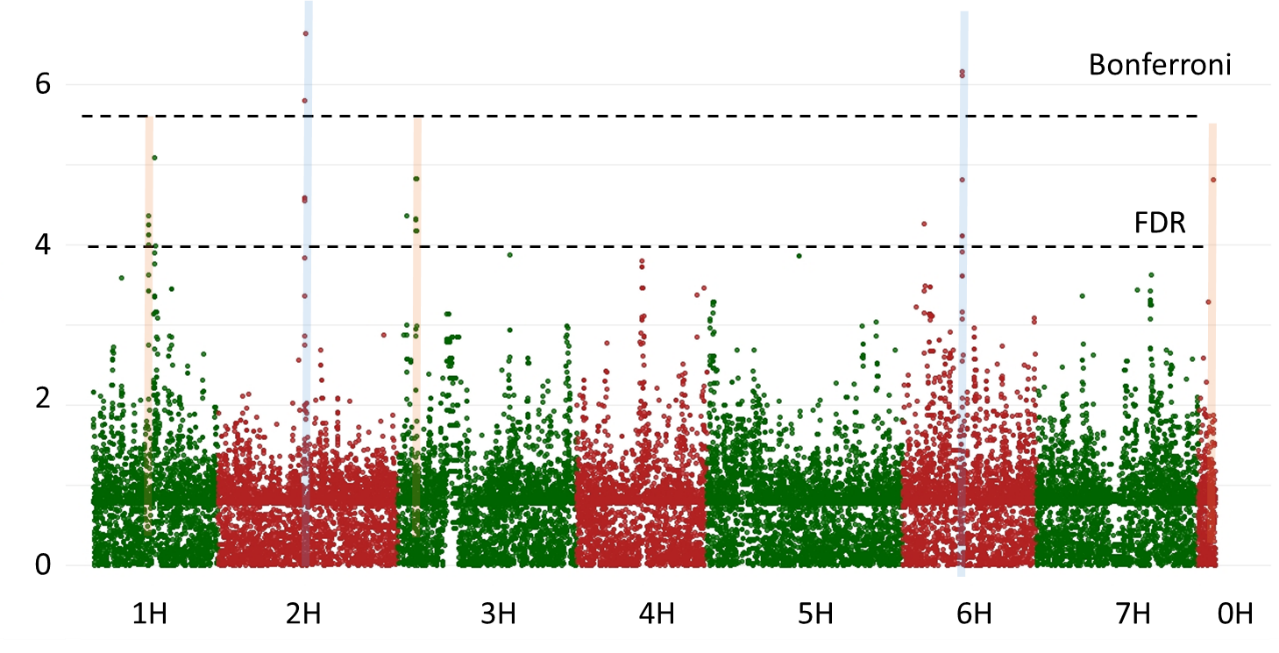


**GLM+PCA+Q model for the isolate K5.1:**


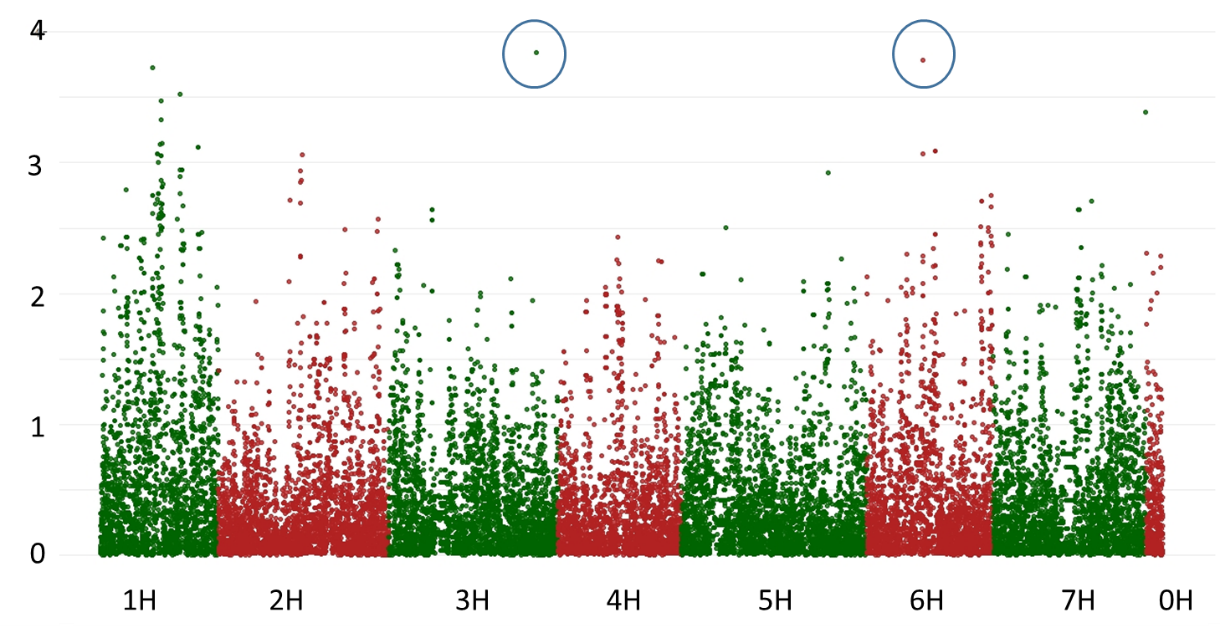


**GLM+PCA+Q model for the isolate S10.2:**


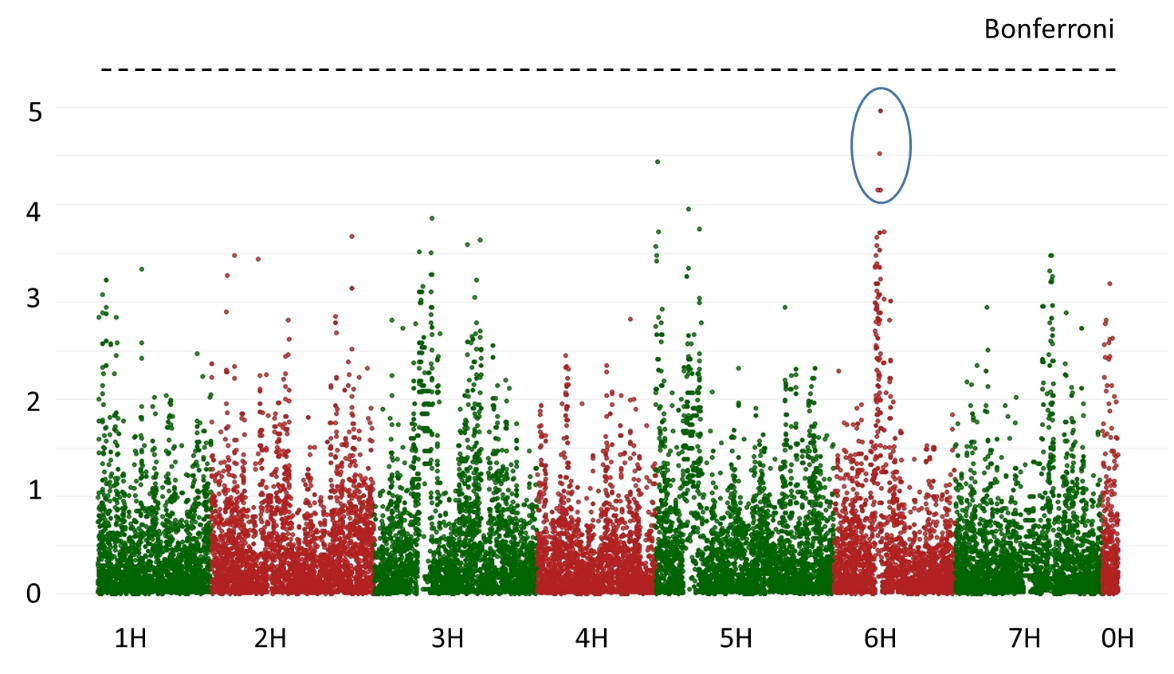

Supplement: Supplementary file 3 — Association mapping results using different models: GLM + Q (GLM + Q-matrix to account for population structure), GLM + PCA, GLM + PCA + Q, MLM + K (MLM with kinship matrix). Dash line named “Bonferroni” corresponds the Bonferroni threshold. Dash line named “FDR” corresponds the FDR (false discovered rate) threshold. (DOCX 5895 kb) [file 12864_2019_5623_MOESM3_ESM.docx]
